# Supplementary material for: Are the shoulder joint function, stability, and mobility tests predictive of handstand execution?
Source: PLoS One. 2024 May 13;19(5):e0302922. doi: 10.1371/journal.pone.0302922 (PMC11090318; doi:10.1371/journal.pone.0302922)
Supplement: S1 Checklist — (DOC) [file pone.0302922.s001.doc]

STROBE Statement—Checklist of items that should be included in reports of ***cross-sectional studies***

|  | Item No | Recommendation |
| --- | --- | --- |
| **Title and abstract** | 1 | (*a*) Indicate the study’s design with a commonly used term in the title or the abstract Are the shoulder joint function, stability, and mobility tests predictive of handstand execution? (Page 1; Cross-sectional study) |
|  |  | (*b*) Provide in the abstract an informative and balanced summary of what was done and what was found  Page 2; Lines 2-18 |
| Introduction | | |
| Background/rationale | 2 | Explain the scientific background and rationale for the investigation being reported Page 3-5 |
| Objectives | 3 | State specific objectives, including any prespecified hypotheses  Page 5; Lines 8-12 |
| Methods | | |
| Study design | 4 | Present key elements of study design early in the paper  Cross-sectional study |
| Setting | 5 | Describe the setting, locations, and relevant dates, including periods of recruitment, exposure, follow-up, and data collection  Page 5-12 |
| Participants | 6 | Give the eligibility criteria, and the sources and methods of selection of participants  Page 5-6; Lines 20-22; 1-10 |
| Variables | 7 | Clearly define all outcomes, exposures, predictors, potential confounders, and effect modifiers. Give diagnostic criteria, if applicable  Page 12, Lines 8-18 |
| Data sources/ measurement | 8* | For each variable of interest, give sources of data and details of methods of assessment (measurement). Describe comparability of assessment methods if there is more than one group  Page 6-12; No group separation |
| Bias | 9 | Describe any efforts to address potential sources of bias  Evaluation of the quality of handstand execution – Page 9; Lines 7-10 |
| Study size | 10 | Explain how the study size was arrived at.  Not applicate |
| Quantitative variables | 11 | Explain how quantitative variables were handled in the analyses. If applicable, describe which groupings were chosen and why  Page 10-12 |
| Statistical methods | 12 | (*a*) Describe all statistical methods, including those used to control for confounding Page 10-12 |
|  |  | (*b*) Describe any methods used to examine subgroups and interactions  Not applicable |
|  |  | (*c*) Explain how missing data were addressed  Participants with any missing data were removed |
|  |  | (*d*) If applicable, describe analytical methods taking account of sampling strategy The sample size was justified by size and time constraint |
|  |  | (*e*) Describe any sensitivity analyses  Not applicate |
| Results | | |
| Participants | 13* | Report numbers of individuals at each stage of study—eg numbers potentially eligible, examined for eligibility, confirmed eligible, included in the study, completing follow-up, and analysed  Page 5; Line 21 |
|  |  | Give reasons for non-participation at each stage  Any missing data; one participant was removed due to outlier age (111 of 125 original) |
|  |  | (c) Consider use of a flow diagram  Not applicate |
| Descriptive data | 14* | Give characteristics of study participants (eg demographic, clinical, social) and information on exposures and potential confounders  Page 5-6; Lines 20-22; 1-10 |
|  |  | (b) Indicate number of participants with missing data for each variable of interest 125 participants (14 removed; 111 recruited), 13 participants were removed due to incomplete data, 1 was removed due to age outlier |
| Outcome data | 15* | Report numbers of outcome events or summary measures  Page 13-15, Table 1 |
| Main results | 16 | Give unadjusted estimates and, if applicable, confounder-adjusted estimates and their precision (eg, 95% confidence interval). Make clear which confounders were adjusted for and why they were included  Page 13-15, Table 2, 3 |
|  |  | (*b*) Report category boundaries when continuous variables were categorized |
|  |  | (*c*) If relevant, consider translating estimates of relative risk into absolute risk for a meaningful time period  Page 13-15, Table 2, 3 |
| Other analyses | 17 | Report other analyses done—eg analyses of subgroups and interactions, and sensitivity analyses  Not applicable |
| Discussion | | |
| Key results | 18 | Summarise key results with reference to study objectives  Page 16, Lines 1-12 |
| Limitations | 19 | Discuss limitations of the study, taking into account sources of potential bias or imprecision. Discuss both direction and magnitude of any potential bias  Page 16-17; Lines 22-25; 1-18 |
| Interpretation | 20 | Give a cautious overall interpretation of results considering objectives, limitations, multiplicity of analyses, results from similar studies, and other relevant evidence Page 15-18 |
| Generalisability | 21 | Discuss the generalisability (external validity) of the study results  Data collection and analysis was realised by standard methods and procedures, and described in detail within methods of the study |
| Other information | | |
| Funding | 22 | Give the source of funding and the role of the funders for the present study and, if applicable, for the original study on which the present article is based  Page 19; Lines 5-6 |

*Give information separately for exposed and unexposed groups.

**Note:** An Explanation and Elaboration article discusses each checklist item and gives methodological background and published examples of transparent reporting. The STROBE checklist is best used in conjunction with this article (freely available on the Web sites of PLoS Medicine at http://www.plosmedicine.org/, Annals of Internal Medicine at http://www.annals.org/, and Epidemiology at http://www.epidem.com/). Information on the STROBE Initiative is available at www.strobe-statement.org.
