# Supplementary material for: Are the shoulder joint function, stability, and mobility tests predictive of handstand execution?
Source: PLoS One. 2024 May 13;19(5):e0302922. doi: 10.1371/journal.pone.0302922 (PMC11090318; doi:10.1371/journal.pone.0302922)
Supplement: S2 File — (HTML) [file pone.0302922.s003.html]

Are the shoulder joint function, stability, and mobility tests predictive of handstand execution? A complete data analysis


# Are the shoulder joint function, stability, and mobility tests predictive of handstand execution? A complete data analysis

2023-12-15

Roman Malíř

- Libraries needed for data
  analysis
  - Summary table of
    individual libraries.
- Data loading and
  preparation
- Visualization of the data
  - Visualization of
    handstand evaluation.
- Summary
  statistics
- Concordance between
  evaluators
  - Kendall’s
    coefficient of concordance between raters in AQV.
  - Kendall’s
    coefficient of concordance also with 95% onfidence intervals.
  - Kendall’s
    coefficient of concordance between raters in E-score.
  - Kendall’s
    coefficient of concordance also with 95% confidence intervals.
- Correlation between the
  data
- Quality
  of handstand execution in relation to shoulder function tests
  - General assumptions of the
    models
  - Model 1: AQV ~ UQYBT +
    CKCUEST
  - Model 2: E-score ~ UQYBT +
    CKCUEST
  - Plot of
    proportional odds ratios (PORs) with error bars.

# Libraries needed for data analysis

```
library(readxl)
library(writexl)
library(ggplot2)
library(ggpubr)
library(gridExtra)
library(grid)
library(pastecs)
library(dplyr)
library(irr)
library(rcompanion)
library(car)
library(foreign)
library(MASS)
library(Hmisc)
library(reshape2)
library(brant)
library(performance)
library(effects)
library(splines)
library(lmtest)
library(knitr)
library(NSM3)
```

### Summary table of individual libraries.

##### *We used the `write_excel` and `read_excel` functions from the `writexl`1 and `readxl`2 packages to create the single library lookup table.*

```
print(packages)
```

```
## # A tibble: 21 x 3
##    Package    Description                                                Version
##    <chr>      <chr>                                                      <chr>  
##  1 writexl    "Export Data Frames to Excel 'xlsx' Format"                1.4.2  
##  2 readxl     "Read Excel Files"                                         1.4.3  
##  3 ggplot2    "Create Elegant Data Visualisations Using the Grammar of ~ 3.4.4  
##  4 ggpubr     "'ggplot2' Based Publication Ready Plots"                  0.6.0  
##  5 gridExtra  "Miscellaneous Functions for \"Grid\" Graphics"            2.3    
##  6 grid       "The Grid Graphics Package"                                4.3.2  
##  7 pastecs    "Package for Analysis of Space-Time Ecological Series"     1.3.21 
##  8 irr        "Various Coefficients of Interrater Reliability and Agree~ 0.84.1 
##  9 dplyr      "A Grammar of Data Manipulation"                           1.1.4  
## 10 rcompanion "Functions to Support Extension Education Program Evaluat~ 2.4.34 
## # i 11 more rows
```

# Data loading and preparation

```
setwd("C:/Users/malir/Desktop")
handstandART <- readxl::read_excel('Supplemental_online_material_raw_dataset.xlsx')
head(handstandART)
```

```
## # A tibble: 6 x 25
##   sex     age height weight sport         laterality_arm laterality_leg `pain_%`
##   <chr> <dbl>  <dbl>  <dbl> <chr>                  <dbl>          <dbl>    <dbl>
## 1 M        19   161    56.3 other sports               1              1     0.36
## 2 F        19   173.   67.9 other sports               1              1     0.26
## 3 M        20   190.   91.8 ice hockey                 1              1     0.34
## 4 M        20   183    87.9 ice hockey                 1              1     0.64
## 5 M        20   176.   62.1 soccer                     1              1     0.36
## 6 M        21   174.   68.5 other sports~              1              1     0.4 
## # i 17 more variables: `trouble_%` <dbl>, `full_score_%` <dbl>, scale_1 <dbl>,
## #   E_score_1 <dbl>, scale_2 <dbl>, E_score_2 <dbl>, scale_3 <dbl>,
## #   E_score_3 <dbl>, arm_R <dbl>, UQYBTR <dbl>, arm_L <dbl>, UQYBTL <dbl>,
## #   CKCUEST <dbl>, SPT <dbl>, UQYBT <dbl>, AQV <dbl>, E_score <dbl>
```

# Visualization of the data

### Visualization of handstand evaluation.

##### *The frequency of occurrence of the measured values of the dependent variable (AQV and E-score) can be seen below. The visualization was performed using the `ggplot2`3, `ggpubr`4, `grid`5, and `gridExtra`6 packages.*

```
AQV_count <- ggplot2::ggplot(handstandART, aes(x = AQV)) + geom_bar(fill = 'darkgrey')+
  xlab('AQV')+
  ylab('Frequency')+
  theme(axis.text.x = element_text(size=10, hjust = 1),
        axis.text.y = element_text(size=10, hjust = 1),
        axis.title.x = element_text(size=20, face = 'bold'),
        axis.title.y = element_text(size=20, face = 'bold'))+
  theme(plot.margin = margin(3,1,1,1, "cm"),
        panel.grid.major = element_blank(), panel.grid.minor = element_blank(),
        panel.background = element_blank(), axis.line = element_line(colour = "black"))

Escore_count <- ggplot2::ggplot(handstandART, aes(x = E_score)) + geom_bar(fill = 'darkgrey')+
  xlab('E-score')+
  ylab('Frequency')+
  theme(axis.text.x = element_text(size=10, hjust = 1),
        axis.text.y = element_text(size=10, hjust = 1),
        axis.title.x = element_text(size=20, face = 'bold'),
        axis.title.y = element_text(size=20, face = 'bold'))+
  theme(plot.margin = margin(3,1,1,0.5, "cm"),
        panel.grid.major = element_blank(), panel.grid.minor = element_blank(),
        panel.background = element_blank(), axis.line = element_line(colour = "black"))+
  xlim(0, 2.4)

grid.arrange(arrangeGrob(AQV_count, Escore_count, ncol = 2, nrow = 1,
                         top = textGrob("",
                             gp = gpar(fontsize = 20, font = 3),
                             hjust = 0.5, vjust = 3)))
```

# Summary statistics

##### *For descriptive statistics we used the `stat.desc` function from the `pastecs`7 package.*

```
head(stat.desc(handstandART))
```

```
##          sex age height weight sport laterality_arm laterality_leg pain_%
## nbr.val   NA 111  111.0  111.0    NA            111            111 111.00
## nbr.null  NA   0    0.0    0.0    NA              7             30  57.00
## nbr.na    NA   0    0.0    0.0    NA              0              0   0.00
## min       NA  19  152.1   43.5    NA              0              0   0.00
## max       NA  23  202.0   91.8    NA              2              2   0.72
## range     NA   4   49.9   48.3    NA              2              2   0.72
##          trouble_% full_score_% scale_1 E_score_1 scale_2 E_score_2 scale_3
## nbr.val   111.0000        111.0     111     111.0     111     111.0     111
## nbr.null   42.0000         33.0      20       1.0      23       1.0      21
## nbr.na      0.0000          0.0       0       0.0       0       0.0       0
## min         0.0000          0.0       0       0.0       0       0.0       0
## max         0.1625          0.3       4       2.4       4       2.1       4
## range       0.1625          0.3       4       2.4       4       2.1       4
##          E_score_3 arm_R UQYBTR arm_L UQYBTL CKCUEST SPT UQYBT AQV E_score
## nbr.val      111.0   111  111.0   111  111.0 111.000 111 111.0 111   111.0
## nbr.null       1.0     0    0.0     0    0.0   0.000   0   0.0  21     0.0
## nbr.na         0.0     0    0.0     0    0.0   0.000   0   0.0   0     0.0
## min            0.0    75   70.2    77   73.4  15.665   1  71.8   0     0.1
## max            2.6   101  100.0   101  100.8  39.330   3 100.4   4     2.2
## range          2.6    26   29.8    24   27.4  23.665   2  28.6   4     2.1
```

##### *To better estimate the central tendency of the variables used in the following analysis, we used the median in addition to the mean (we used the `summarise` function from the `dplyr`8 package).*

```
median_table <- handstandART %>%
  summarise(
    Median_UQYBTL = median(UQYBTL, na.rm = TRUE),
    Median_UQYBTR = median(UQYBTR, na.rm = TRUE),
    Median_UQYBT = median(UQYBT, na.rm = TRUE),
    Median_CKCUEST = median(CKCUEST, na.rm = TRUE),
    Median_E_score = median(E_score, na.rm = TRUE))
print(median_table)
```

```
## # A tibble: 1 x 5
##   Median_UQYBTL Median_UQYBTR Median_UQYBT Median_CKCUEST Median_E_score
##           <dbl>         <dbl>        <dbl>          <dbl>          <dbl>
## 1          85.9          86.5         86.5           27.8            1.9
```

# Concordance between evaluators

### Kendall’s coefficient of concordance between raters in AQV.

```
(kendall_W_AQV <- irr::kendall(handstandART[c(11,13,15)], correct = T))
```

```
##  Kendall's coefficient of concordance Wt
## 
##  Subjects = 111 
##    Raters = 3 
##        Wt = 0.96 
## 
## Chisq(110) = 317 
##   p-value = 6.24e-22
```

### Kendall’s coefficient of concordance also with 95% onfidence intervals.

```
rcompanion::kendallW(handstandART[c(11,13,15)], correct = T, ci = T)
```

```
##      W lower.ci upper.ci
## 1 0.96     0.96        1
```

### Kendall’s coefficient of concordance between raters in E-score.

```
(kendall_W_Escore <- irr::kendall(handstandART[c(12,14,16)], correct = T))
```

```
##  Kendall's coefficient of concordance Wt
## 
##  Subjects = 111 
##    Raters = 3 
##        Wt = 0.84 
## 
## Chisq(110) = 277 
##   p-value = 2.04e-16
```

### Kendall’s coefficient of concordance also with 95% confidence intervals.

```
rcompanion::kendallW(handstandART[c(12,14,16)], correct = T, ci = T)
```

```
##      W lower.ci upper.ci
## 1 0.84     0.84        1
```

# Correlation between the data

#### Correlation between UQYBTR and UQYBTL.

```
cor.test(handstandART$UQYBTL, handstandART$UQYBTR, method = 'pearson', alternative = "two.sided")
```

```
## 
##  Pearson's product-moment correlation
## 
## data:  handstandART$UQYBTL and handstandART$UQYBTR
## t = 13.08, df = 109, p-value < 2.2e-16
## alternative hypothesis: true correlation is not equal to 0
## 95 percent confidence interval:
##  0.6966624 0.8448788
## sample estimates:
##       cor 
## 0.7815666
```

#### Correlation between UQYBTR and UQYBTT.

```
cor.test(handstandART$UQYBTR, handstandART$UQYBT, method = 'pearson', alternative = "two.sided")
```

```
## 
##  Pearson's product-moment correlation
## 
## data:  handstandART$UQYBTR and handstandART$UQYBT
## t = 30.122, df = 109, p-value < 2.2e-16
## alternative hypothesis: true correlation is not equal to 0
## 95 percent confidence interval:
##  0.9205943 0.9618535
## sample estimates:
##      cor 
## 0.944857
```

#### Correlation between UQYBTL and UQYBTT.

```
cor.test(handstandART$UQYBTL, handstandART$UQYBT, method = 'pearson', alternative = "two.sided")
```

```
## 
##  Pearson's product-moment correlation
## 
## data:  handstandART$UQYBTL and handstandART$UQYBT
## t = 29.516, df = 109, p-value < 2.2e-16
## alternative hypothesis: true correlation is not equal to 0
## 95 percent confidence interval:
##  0.9176133 0.9603895
## sample estimates:
##       cor 
## 0.9427598
```

#### Correlation between UQYBT and CKCUEST.

```
cor.test(handstandART$UQYBT, handstandART$CKCUEST, method = 'pearson', alternative = "two.sided")
```

```
## 
##  Pearson's product-moment correlation
## 
## data:  handstandART$UQYBT and handstandART$CKCUEST
## t = -0.55098, df = 109, p-value = 0.5828
## alternative hypothesis: true correlation is not equal to 0
## 95 percent confidence interval:
##  -0.2367679  0.1350184
## sample estimates:
##        cor 
## -0.0527008
```

#### Correlation between AQV and E-score.

```
cor.test(handstandART$AQV, handstandART$E_score, method = "kendall", conf.level = 0.95)
```

```
## 
##  Kendall's rank correlation tau
## 
## data:  handstandART$AQV and handstandART$E_score
## z = -8.7627, p-value < 2.2e-16
## alternative hypothesis: true tau is not equal to 0
## sample estimates:
##        tau 
## -0.6761623
```

#### Confidence intervals of correlations between AQV and E-score.

```
kendall.ci(handstandART$AQV, handstandART$E_score, alpha = 0.05, type = "t")
```

```
## 
## 1 - alpha = 0.95 two-sided CI for tau:
## -0.772, -0.581
```

##### *Plots of correlations.*

```
G1 <- ggplot2::ggplot(handstandART, mapping = aes(UQYBTR, UQYBTL)) + 
  geom_point(pch = 21, col = 'blue',bg="red", lwd=2, size = 5) +
  geom_smooth(method = "lm", se = FALSE) + 
  theme(panel.grid.major = element_blank(),
        panel.grid.minor = element_blank(),
        panel.background = element_blank())+ 
  theme(axis.text.x = element_text(size=10, hjust = 1),
        axis.text.y = element_text(size=10, hjust = 1),
        axis.title.x = element_text(size=20, face = 'bold'),
        axis.title.y = element_text(size=20, face = 'bold'))

G2 <- ggplot2::ggplot(handstandART, mapping = aes(UQYBTR, UQYBT)) + 
  geom_point(pch = 21, col = 'blue',bg="red", lwd=2, size = 5) +
  geom_smooth(method = "lm", se = FALSE) + 
  theme(panel.grid.major = element_blank(),
        panel.grid.minor = element_blank(),
        panel.background = element_blank())+ 
  theme(axis.text.x = element_text(size=10, hjust = 1),
        axis.text.y = element_text(size=10, hjust = 1),
        axis.title.x = element_text(size=20, face = 'bold'),
        axis.title.y = element_text(size=20, face = 'bold'))

G3 <- ggplot2::ggplot(handstandART, mapping = aes(UQYBTL, UQYBT)) + 
  geom_point(pch = 21, col = 'blue',bg="red", lwd=2, size = 5) +
  geom_smooth(method = "lm", se = FALSE) + 
  theme(panel.grid.major = element_blank(),
        panel.grid.minor = element_blank(),
        panel.background = element_blank())+ 
  theme(axis.text.x = element_text(size=10, hjust = 1),
        axis.text.y = element_text(size=10, hjust = 1),
        axis.title.x = element_text(size=20, face = 'bold'),
        axis.title.y = element_text(size=20, face = 'bold'))

G4 <- ggplot2::ggplot(handstandART, mapping = aes(UQYBT, CKCUEST)) + 
  geom_point(pch = 21, col = 'blue',bg="red", lwd=2, size = 5) +
  geom_smooth(method = "lm", se = FALSE) + 
  theme(panel.grid.major = element_blank(),
        panel.grid.minor = element_blank(),
        panel.background = element_blank())+ 
  theme(axis.text.x = element_text(size=10, hjust = 1),
        axis.text.y = element_text(size=10, hjust = 1),
        axis.title.x = element_text(size=20, face = 'bold'),
        axis.title.y = element_text(size=20, face = 'bold'))

G5 <- ggplot2::ggplot(handstandART, mapping = aes(AQV, E_score)) + 
  geom_point(pch = 21, col = 'blue',bg="red", lwd=2, size = 5)+ 
  geom_smooth(method = "auto", se = F) + 
  theme(panel.grid.major = element_blank(),
        panel.grid.minor = element_blank(),
        panel.background = element_blank())+ 
  theme(axis.text.x = element_text(size=10, hjust = 1),
        axis.text.y = element_text(size=10, hjust = 1),
        axis.title.x = element_text(size=20, face = 'bold'),
        axis.title.y = element_text(size=20, face = 'bold'))
```

##### *Grid of correlations’ visualization.*

```
grid.arrange(G1, G2, G3, G4, G5,
             ncol = 2, nrow = 3,
             top = textGrob("Title",
                            gp = gpar(fontsize = 20, font = 3),
                            hjust = 0.5, vjust = 1),
             heights = c(4, 4, 4),
             widths = c(4, 4))
```

# Quality of handstand execution in relation to shoulder function tests

### General assumptions of the models

#### Variance equality checking (UQYBT ~ E\_score).

```
stats::fligner.test(UQYBT ~ E_score, data = handstandART) # variances are homogeneous
```

```
## 
##  Fligner-Killeen test of homogeneity of variances
## 
## data:  UQYBT by E_score
## Fligner-Killeen:med chi-squared = 23.086, df = 20, p-value = 0.2846
```

#### Variance equality checking (CKCUEST ~ E\_score).

```
stats::fligner.test(CKCUEST ~ E_score, data = handstandART) # variances are homogeneous
```

```
## 
##  Fligner-Killeen test of homogeneity of variances
## 
## data:  CKCUEST by E_score
## Fligner-Killeen:med chi-squared = 27.771, df = 20, p-value = 0.1149
```

#### Checking multicollinearity between all predictors (UQYBT/CKCUEST).

##### If the variance inflation factor (VIF) is greater than 5, multicollinearity is present. If multicollinearity is present, it depends on the order of predictors. We used `vif` function from the `car`9 package.

```
model_001i_vif <- lm(scale(AQV) ~ UQYBT + CKCUEST, data = handstandART)
(VIF_001i <- car::vif(model_001i_vif))
```

```
##    UQYBT  CKCUEST 
## 1.002785 1.002785
```

```
barplot(VIF_001i, main = 'VIF values', horiz = T, col = 'orange', xlim = c(0,7) )
```

### Model 1: AQV ~ UQYBT + CKCUEST

##### *Converting the AQV into factor variable.*

```
handstandART$AQV <- as.factor(handstandART$AQV)
```

##### *Checking the class of all variables.*

```
str(handstandART)
```

```
## tibble [111 x 25] (S3: tbl_df/tbl/data.frame)
##  $ sex           : chr [1:111] "M" "F" "M" "M" ...
##  $ age           : num [1:111] 19 19 20 20 20 21 20 21 20 21 ...
##  $ height        : num [1:111] 161 173 190 183 176 ...
##  $ weight        : num [1:111] 56.3 67.9 91.8 87.9 62.1 68.5 57.8 80.1 71 78.5 ...
##  $ sport         : chr [1:111] "other sports" "other sports" "ice hockey" "ice hockey" ...
##  $ laterality_arm: num [1:111] 1 1 1 1 1 1 0 1 1 1 ...
##  $ laterality_leg: num [1:111] 1 1 1 1 1 1 0 1 1 1 ...
##  $ pain_%        : num [1:111] 0.36 0.26 0.34 0.64 0.36 0.4 0.52 0.72 0.6 0.65 ...
##  $ trouble_%     : num [1:111] 0.1375 0.1125 0.075 0.025 0.0125 ...
##  $ full_score_%  : num [1:111] 0.223 0.169 0.176 0.262 0.146 ...
##  $ scale_1       : num [1:111] 1 0 0 1 1 0 2 1 1 1 ...
##  $ E_score_1     : num [1:111] 1.9 2.1 1.9 2.1 2.1 2.1 1.9 2 2 1.9 ...
##  $ scale_2       : num [1:111] 1 0 0 1 1 0 2 1 1 0 ...
##  $ E_score_2     : num [1:111] 1.9 2 2 1.6 1.9 2 1.4 1.8 1.8 2 ...
##  $ scale_3       : num [1:111] 0 0 0 1 1 0 2 1 1 0 ...
##  $ E_score_3     : num [1:111] 0 2.1 2.2 1.8 2 2.1 1.7 1.9 1.9 1.8 ...
##  $ arm_R         : num [1:111] 82.5 86 95 91 89 89 86 90 92 92.5 ...
##  $ UQYBTR        : num [1:111] 83.6 77.1 90.2 89.4 93.3 86.9 86.4 88.1 80.1 78.6 ...
##  $ arm_L         : num [1:111] 82 85 96 90 90.5 86.5 86 91.5 92.5 93 ...
##  $ UQYBTL        : num [1:111] 87.8 77.6 86.5 89.3 93.2 90.9 89.5 86.7 80.7 74.6 ...
##  $ CKCUEST       : num [1:111] 29.7 23.3 30 39.3 36.3 ...
##  $ SPT           : num [1:111] 2 3 2 2 2 2 2 2 2 2 ...
##  $ UQYBT         : num [1:111] 85.7 77.3 88.3 89.3 93.2 ...
##  $ AQV           : Factor w/ 5 levels "0","1","2","3",..: 2 1 1 2 2 1 3 2 2 1 ...
##  $ E_score       : num [1:111] 1.3 2.1 2 1.8 2 2.1 1.7 1.9 1.9 1.9 ...
```

##### *Formula for the ordinal logistic regression model (Model 1).*

```
formula_1 <- as.formula(AQV ~ UQYBT + CKCUEST)
```

#### Making the model of ordinal logistic regression using `polr` function from the `MASS`10 package.

```
model_001i <- MASS::polr(formula_1, data = handstandART, Hess = T, method = "logistic")
```

##### *Printing model information.*

```
print(model_001i)
```

```
## Call:
## MASS::polr(formula = formula_1, data = handstandART, Hess = T, 
##     method = "logistic")
## 
## Coefficients:
##       UQYBT     CKCUEST 
##  0.04684879 -0.01341928 
## 
## Intercepts:
##      0|1      1|2      2|3      3|4 
## 2.176357 5.013229 6.237230 6.969416 
## 
## Residual Deviance: 249.0097 
## AIC: 261.0097
```

```
summary(model_001i)
```

```
## Call:
## MASS::polr(formula = formula_1, data = handstandART, Hess = T, 
##     method = "logistic")
## 
## Coefficients:
##            Value Std. Error t value
## UQYBT    0.04685    0.03615  1.2960
## CKCUEST -0.01342    0.04931 -0.2721
## 
## Intercepts:
##     Value   Std. Error t value
## 0|1  2.1764  3.4279     0.6349
## 1|2  5.0132  3.4637     1.4474
## 2|3  6.2372  3.4842     1.7901
## 3|4  6.9694  3.5055     1.9881
## 
## Residual Deviance: 249.0097 
## AIC: 261.0097
```

##### *Next, we examine the estimates for the two intercepts, also referred to as cut-points. These intercepts signify the points at which the continuous latent variable is divided to form the five groups observed in our data. It is important to note that the latent variable itself is continuous. Typically, these intercepts are not employed in interpreting the results. The cut-points are intricately connected to thresholds, which are reported by other statistical packages.*

#### Making a table with coefficients and cut-points, t values, and p values of the Model 1.

```
coef_model_001i <- coef(summary(model_001i))
p_model_001i <- pnorm(abs(coef_model_001i[, "t value"]), lower.tail = FALSE) * 2
coef_model_001i <- cbind(coef_model_001i, 'p value' = p_model_001i)
print(coef_model_001i)
```

```
##               Value Std. Error    t value    p value
## UQYBT    0.04684879 0.03614781  1.2960340 0.19496377
## CKCUEST -0.01341928 0.04930868 -0.2721484 0.78550791
## 0|1      2.17635726 3.42793424  0.6348889 0.52550090
## 1|2      5.01322860 3.46368827  1.4473671 0.14779412
## 2|3      6.23722993 3.48422616  1.7901335 0.07343245
## 3|4      6.96941556 3.50548463  1.9881461 0.04679553
```

#### Adjusted R squared calculation of the Model 1 (we used `r2_mcfadden` function from the `performance`11 package).

```
r2_mcfadden(model_001i)
```

```
## # R2 for Generalized Linear Regression
##        R2: 0.007
##   adj. R2: -0.001
```

#### Chi squared calculation of the Model 1 (we used `Anova` function from the `car`12 package).

```
Anova(model_001i)
```

```
## Analysis of Deviance Table (Type II tests)
## 
## Response: AQV
##         LR Chisq Df Pr(>Chisq)
## UQYBT    1.68283  1     0.1945
## CKCUEST  0.07398  1     0.7856
```

#### 95% CIs of the Model 1.

```
confint(model_001i)
```

```
##               2.5 %     97.5 %
## UQYBT   -0.02396151 0.11812231
## CKCUEST -0.11061038 0.08324014
```

#### Coefficients of the Model 1.

```
model_001i$coefficients
```

```
##       UQYBT     CKCUEST 
##  0.04684879 -0.01341928
```

#### Exponential of the coefficients of the Model 1.

```
exp(coef(model_001i))
```

```
##     UQYBT   CKCUEST 
## 1.0479635 0.9866704
```

##### *Ensuring the validity of the ‘proportional odds assumption’ or the ‘parallel regression assumption’ involves assuming that the coefficients describing the relationship between, for example, the lowest category versus all higher categories of the dependent variable are the same as those describing the relationship between the next lowest category and all higher categories, and so on. The Brant’s test aids in confirming the validity of the parallel assumption by indicating that the probabilities (p-values) for all variables exceed the threshold of alpha = 0.05. Additionally, the output includes an Omnibus variable, representing the entire model, which must still have a p-value greater than 0.05.*

#### Parallel regression assumption and the Brant’s test of the Model 1.

```
Brant_model_001i <-brant(model_001i)
```

```
## -------------------------------------------- 
## Test for X2  df  probability 
## -------------------------------------------- 
## Omnibus      2.27    6   0.89
## UQYBT        1.1 3   0.78
## CKCUEST      1.39    3   0.71
## -------------------------------------------- 
## 
## H0: Parallel Regression Assumption holds
```

```
print(Brant_model_001i)
```

```
##               X2 df probability
## Omnibus 2.274861  6   0.8927646
## UQYBT   1.104022  3   0.7761032
## CKCUEST 1.385774  3   0.7088728
```

### Model 2: E-score ~ UQYBT + CKCUEST

##### *Converting the AQV into factor variable.*

```
handstandART$E_score <- as.factor(handstandART$E_score)
```

##### *Checking the class of all variables.*

```
str(handstandART)
```

```
## tibble [111 x 25] (S3: tbl_df/tbl/data.frame)
##  $ sex           : chr [1:111] "M" "F" "M" "M" ...
##  $ age           : num [1:111] 19 19 20 20 20 21 20 21 20 21 ...
##  $ height        : num [1:111] 161 173 190 183 176 ...
##  $ weight        : num [1:111] 56.3 67.9 91.8 87.9 62.1 68.5 57.8 80.1 71 78.5 ...
##  $ sport         : chr [1:111] "other sports" "other sports" "ice hockey" "ice hockey" ...
##  $ laterality_arm: num [1:111] 1 1 1 1 1 1 0 1 1 1 ...
##  $ laterality_leg: num [1:111] 1 1 1 1 1 1 0 1 1 1 ...
##  $ pain_%        : num [1:111] 0.36 0.26 0.34 0.64 0.36 0.4 0.52 0.72 0.6 0.65 ...
##  $ trouble_%     : num [1:111] 0.1375 0.1125 0.075 0.025 0.0125 ...
##  $ full_score_%  : num [1:111] 0.223 0.169 0.176 0.262 0.146 ...
##  $ scale_1       : num [1:111] 1 0 0 1 1 0 2 1 1 1 ...
##  $ E_score_1     : num [1:111] 1.9 2.1 1.9 2.1 2.1 2.1 1.9 2 2 1.9 ...
##  $ scale_2       : num [1:111] 1 0 0 1 1 0 2 1 1 0 ...
##  $ E_score_2     : num [1:111] 1.9 2 2 1.6 1.9 2 1.4 1.8 1.8 2 ...
##  $ scale_3       : num [1:111] 0 0 0 1 1 0 2 1 1 0 ...
##  $ E_score_3     : num [1:111] 0 2.1 2.2 1.8 2 2.1 1.7 1.9 1.9 1.8 ...
##  $ arm_R         : num [1:111] 82.5 86 95 91 89 89 86 90 92 92.5 ...
##  $ UQYBTR        : num [1:111] 83.6 77.1 90.2 89.4 93.3 86.9 86.4 88.1 80.1 78.6 ...
##  $ arm_L         : num [1:111] 82 85 96 90 90.5 86.5 86 91.5 92.5 93 ...
##  $ UQYBTL        : num [1:111] 87.8 77.6 86.5 89.3 93.2 90.9 89.5 86.7 80.7 74.6 ...
##  $ CKCUEST       : num [1:111] 29.7 23.3 30 39.3 36.3 ...
##  $ SPT           : num [1:111] 2 3 2 2 2 2 2 2 2 2 ...
##  $ UQYBT         : num [1:111] 85.7 77.3 88.3 89.3 93.2 ...
##  $ AQV           : Factor w/ 5 levels "0","1","2","3",..: 2 1 1 2 2 1 3 2 2 1 ...
##  $ E_score       : Factor w/ 21 levels "0.1","0.2","0.3",..: 12 20 19 17 19 20 16 18 18 18 ...
```

##### *Formula for the ordinal logistic regression model (Model 2).*

```
formula_2 <- as.formula(E_score ~ UQYBT + CKCUEST)
```

#### Making the model of ordinal logistic regression.

```
model_002i <- MASS::polr(formula_2, data = handstandART, Hess = T, method = "logistic")
```

##### *Printing model information.*

```
print(model_002i)
```

```
## Call:
## MASS::polr(formula = formula_2, data = handstandART, Hess = T, 
##     method = "logistic")
## 
## Coefficients:
##         UQYBT       CKCUEST 
## -0.0359739084  0.0003735299 
## 
## Intercepts:
##    0.1|0.2    0.2|0.3    0.3|0.4    0.4|0.5    0.5|0.7    0.7|0.8    0.8|0.9 
## -6.6815620 -6.3853944 -6.1521379 -5.7944289 -5.5231140 -5.2043626 -5.1135050 
##      0.9|1      1|1.1    1.1|1.2    1.2|1.3    1.3|1.4    1.4|1.5    1.5|1.6 
## -4.9486413 -4.6682785 -4.5455938 -4.3768160 -4.1735277 -4.1258273 -3.9019497 
##    1.6|1.7    1.7|1.8    1.8|1.9      1.9|2      2|2.1    2.1|2.2 
## -3.6562334 -3.5781813 -3.2059596 -2.3777318 -1.0414190  0.2251825 
## 
## Residual Deviance: 565.5695 
## AIC: 609.5695
```

```
summary(model_002i)
```

```
## Call:
## MASS::polr(formula = formula_2, data = handstandART, Hess = T, 
##     method = "logistic")
## 
## Coefficients:
##              Value Std. Error   t value
## UQYBT   -0.0359739    0.03182 -1.130534
## CKCUEST  0.0003735    0.04529  0.008247
## 
## Intercepts:
##         Value   Std. Error t value
## 0.1|0.2 -6.6816  3.1700    -2.1078
## 0.2|0.3 -6.3854  3.1576    -2.0222
## 0.3|0.4 -6.1521  3.1494    -1.9534
## 0.4|0.5 -5.7944  3.1380    -1.8465
## 0.5|0.7 -5.5231  3.1317    -1.7636
## 0.7|0.8 -5.2044  3.1254    -1.6652
## 0.8|0.9 -5.1135  3.1235    -1.6371
## 0.9|1   -4.9486  3.1206    -1.5858
## 1|1.1   -4.6683  3.1166    -1.4979
## 1.1|1.2 -4.5456  3.1151    -1.4592
## 1.2|1.3 -4.3768  3.1130    -1.4060
## 1.3|1.4 -4.1735  3.1103    -1.3418
## 1.4|1.5 -4.1258  3.1097    -1.3268
## 1.5|1.6 -3.9019  3.1077    -1.2556
## 1.6|1.7 -3.6562  3.1056    -1.1773
## 1.7|1.8 -3.5782  3.1047    -1.1525
## 1.8|1.9 -3.2060  3.1028    -1.0333
## 1.9|2   -2.3777  3.0961    -0.7680
## 2|2.1   -1.0414  3.0943    -0.3366
## 2.1|2.2  0.2252  3.1204     0.0722
## 
## Residual Deviance: 565.5695 
## AIC: 609.5695
```

#### Making a table with coefficients and cut-points, t values, and p values of the Model 2.

```
coef_model_002i <- coef(summary(model_002i))
p_model_002i <- pnorm(abs(coef_model_002i[, "t value"]), lower.tail = FALSE) * 2
coef_model_002i <- cbind(coef_model_002i, 'p value' = p_model_002i)
print(coef_model_002i)
```

```
##                 Value Std. Error      t value    p value
## UQYBT   -0.0359739084 0.03182029 -1.130533581 0.25825146
## CKCUEST  0.0003735299 0.04529078  0.008247371 0.99341962
## 0.1|0.2 -6.6815620359 3.16996997 -2.107768247 0.03505104
## 0.2|0.3 -6.3853944192 3.15763917 -2.022205225 0.04315515
## 0.3|0.4 -6.1521378661 3.14937857 -1.953445017 0.05076689
## 0.4|0.5 -5.7944289049 3.13800239 -1.846534254 0.06481467
## 0.5|0.7 -5.5231140245 3.13167045 -1.763631937 0.07779397
## 0.7|0.8 -5.2043625970 3.12537747 -1.665194892 0.09587388
## 0.8|0.9 -5.1135049666 3.12354347 -1.637084617 0.10161279
## 0.9|1   -4.9486412893 3.12057017 -1.585813176 0.11278170
## 1|1.1   -4.6682785135 3.11659966 -1.497875577 0.13416558
## 1.1|1.2 -4.5455937571 3.11510605 -1.459209956 0.14450733
## 1.2|1.3 -4.3768160490 3.11301233 -1.405974532 0.15973169
## 1.3|1.4 -4.1735277187 3.11030429 -1.341839040 0.17964819
## 1.4|1.5 -4.1258273475 3.10969383 -1.326763202 0.18458703
## 1.5|1.6 -3.9019496948 3.10768106 -1.255582416 0.20926741
## 1.6|1.7 -3.6562334336 3.10555706 -1.177319677 0.23906794
## 1.7|1.8 -3.5781812556 3.10474070 -1.152489564 0.24911996
## 1.8|1.9 -3.2059595990 3.10278187 -1.033253297 0.30148538
## 1.9|2   -2.3777318193 3.09606942 -0.767984014 0.44249668
## 2|2.1   -1.0414189645 3.09432890 -0.336557296 0.73645065
## 2.1|2.2  0.2251825463 3.12038693  0.072164943 0.94247064
```

#### Adjusted R squared calculation of the Model 2.

```
r2_mcfadden(model_002i)
```

```
## # R2 for Generalized Linear Regression
##        R2: 0.002
##   adj. R2: -0.001
```

#### Chi squared calculation of the Model 2.

```
Anova(model_002i)
```

```
## Analysis of Deviance Table (Type II tests)
## 
## Response: E_score
##         LR Chisq Df Pr(>Chisq)
## UQYBT    1.27893  1     0.2581
## CKCUEST  0.00007  1     0.9934
```

#### 95% CIs of the Model 2.

```
confint(model_002i)
```

```
##               2.5 %     97.5 %
## UQYBT   -0.09861066 0.02644602
## CKCUEST -0.08827566 0.09013337
```

#### Coefficients of the Model 2.

```
model_002i$coefficients
```

```
##         UQYBT       CKCUEST 
## -0.0359739084  0.0003735299
```

#### Exponential of the coefficients of the Model 2.

```
exp(coef(model_002i))
```

```
##     UQYBT   CKCUEST 
## 0.9646655 1.0003736
```

#### Parallel regression assumption and the Brant’s test of the Model 2.

```
Brant_model_002i <-brant(model_002i)
```

```
## -------------------------------------------- 
## Test for X2  df  probability 
## -------------------------------------------- 
## Omnibus      52.35   38  0.06
## UQYBT        24.02   19  0.2
## CKCUEST      22.93   19  0.24
## -------------------------------------------- 
## 
## H0: Parallel Regression Assumption holds
```

```
print(Brant_model_002i)
```

```
##               X2 df probability
## Omnibus 52.35340 38  0.06058397
## UQYBT   24.01526 19  0.19556757
## CKCUEST 22.93216 19  0.24034320
```

### Plot of proportional odds ratios (PORs) with error bars.

```
ORcoef1 <- exp(cbind(OR = coef(model_001i), confint(model_001i)))
base_AQV <- data.frame(mean = model_001i$coefficients,
                       ci.lb = c(ORcoef1[,2]),
                       ci.ub = c(ORcoef1[,3]),
                       variable = c("UQYBT on AQV", "CKCUEST on AQV"),
                       POR = c(exp(coef(model_001i))))

ORcoef2 <- exp(cbind(OR = coef(model_002i), confint(model_002i)))
base_Escore <- data.frame(mean = model_002i$coefficients,
                          lower = c(ORcoef2[,2]),
                          upper = c(ORcoef2[,3]),
                          variable = c("UQYBT on E-score", "CKCUEST on E-score"),
                          POR = c(exp(coef(model_002i))))

names(base_AQV)[names(base_AQV) == "ci.lb"] <- "lower"
names(base_AQV)[names(base_AQV) == "ci.ub"] <- "upper"
base <- rbind(base_Escore,base_AQV)

View(base)
rownames <- rownames(base)
rownames(base) <- c("1", "2", "3", "4")
print(base)
```

```
##            mean     lower    upper           variable       POR
## 1 -0.0359739084 0.9060954 1.026799   UQYBT on E-score 0.9646655
## 2  0.0003735299 0.9155085 1.094320 CKCUEST on E-score 1.0003736
## 3  0.0468487898 0.9763233 1.125382       UQYBT on AQV 1.0479635
## 4 -0.0134192782 0.8952875 1.086803     CKCUEST on AQV 0.9866704
```

```
# fix the order of variables
base$variable <- factor(base$variable, levels = base$variable)

# plot contributions of coefficients with CI's
ggplot(base, aes(x = variable, y = POR, ymin = lower, ymax = upper)) +
  geom_point(position = position_dodge(width = 0.2)) +
  geom_errorbar(position = position_dodge(width = 0.2), width = 0.1) +
  coord_flip()+
  theme_classic()+
  scale_y_continuous(limits = c(0.0,3.0))+
  geom_hline(yintercept = 1.0, lwd = 0.2, lty = 2)+
  theme(text = element_text(size=16, family="Comic Sans MS", face = "bold"),plot.margin = margin(2, 2, 2, 1, "cm"),
        axis.ticks.y = element_blank(),
        panel.grid = element_blank(),element_line(linetype = 1),
        axis.line.y = element_blank(),axis.title.x = element_text(vjust = -5, hjust = 0.2),
        legend.position = "none")+
  ylab(expression(paste("<- Decrement   Improvement ->\n    (Proportional odds ratio)")))+
  xlab("")
```

---

1. Ooms J (2023). writexl: Export Data Frames to Excel
   ‘xlsx’ Format. R package version 1.4.2, https://CRAN.R-project.org/package=writexl.↩︎
2. Wickham H, Bryan J (2023). readxl: Read Excel Files. R
   package version 1.4.3, https://CRAN.R-project.org/package=readxl.↩︎
3. H. Wickham. ggplot2: Elegant Graphics for Data Analysis.
   Springer-Verlag New York, 2016.↩︎
4. R Core Team (2023). R: A Language and Environment for
   Statistical Computing. R Foundation for Statistical Computing, Vienna,
   Austria. https://www.R-project.org/.↩︎
5. Auguie B (2017). gridExtra: Miscellaneous Functions for
   “Grid” Graphics. R package version 2.3, https://CRAN.R-project.org/package=gridExtra.↩︎
6. Kassambara A (2023). ggpubr: ‘ggplot2’ Based Publication
   Ready Plots. R package version 0.6.0, https://CRAN.R-project.org/package=ggpubr.↩︎
7. Grosjean P, Ibanez F (2018). pastecs: Package for
   Analysis of Space-Time Ecological Series. R package version 1.3.21, https://CRAN.R-project.org/package=pastecs.↩︎
8. Wickham H, François R, Henry L, Müller K, Vaughan D
   (2023). dplyr: A Grammar of Data Manipulation. R package version 1.1.4,
   https://CRAN.R-project.org/package=dplyr.↩︎
9. Fox J, Weisberg S (2019). *An R Companion to Applied
   Regression*, Third edition. Sage, Thousand Oaks CA. https://socialsciences.mcmaster.ca/jfox/Books/Companion/.↩︎
10. Venables, W. N. & Ripley, B. D. (2002) Modern
    Applied Statistics with S. Fourth Edition. Springer, New York. ISBN
    0-387-95457-0↩︎
11. Lüdecke et al., (2021). performance: An R Package for
    Assessment, Comparison and Testing of Statistical Models. Journal of
    Open Source Software, 6(60), 3139. https://doi.org/10.21105/joss.03139↩︎
12. Fox J, Weisberg S (2019). *An R Companion to Applied
    Regression*, Third edition. Sage, Thousand Oaks CA. https://socialsciences.mcmaster.ca/jfox/Books/Companion/.↩︎
