# Supplementary material for: Are the shoulder joint function, stability, and mobility tests predictive of handstand execution?
Source: PLoS One. 2024 May 13;19(5):e0302922. doi: 10.1371/journal.pone.0302922 (PMC11090318; doi:10.1371/journal.pone.0302922)
Supplement: Attachment — Submitted filename: Rebuttal letter.docx [file pone.0302922.s005.docx]

Dear Dr. Shaheen,

Thank you for sharing your comments and comments from the three reviewers regarding our manuscript submitted to the PlosONE journal. We highly appreciate the insights and believe that this feedback helped us rework our manuscript into a much stronger, better-focused piece. As you have suggested, we are resubmitting the improved version of our manuscript for review to the PlosONE again.

Below are individual comments and related responses (in italics) on how we have amended the manuscript.

# Editor

Providing clear justification for conducting the study with this participant group and a clarity around the types of data collected in the bigger protocol and relevance to this study. The reviewers made a number of comments and suggestions around the suitability of the introduction section and strength of rationale/justification for conducting the study.

*We highly appreciate the comprehensive review of our manuscript. We agree with the raised comments about the original text's length and lack of focus. In the revisited version, we focus on providing a more robust rationale for the study and its sample. We completely reworked the manuscript from top to bottom to reflect these changes.*

*Individual changes across the text are highlighted.*

# Reviewer 1

The Introduction is too long and too many details about the test are provided, this choice generates confusion and gives less focus on the research problem. Delete it.

*Thank you for your comment. We have rearranged and rewrote the Introduction to focus the text better and removed unnecessary details overlapping with the Methods section. Individual changes across the text are highlighted.*

Only 90 minutes of training were attended to learn the handstand, did I understand well?

*All participants underwent one 12-week term of Basics of Gymnastics course focused on learning the fundamentals of gymnastics, including handstands with all necessary drills and preparatory exercises used in learning a static handstand. On top of that, all participants underwent addition two 45-minute lessons directly focused on practicing static handstands. This information is highlighted in the Participants section (see Page 5, lines 21-22 and Page 6, lines 1-9).*

Methods has redundancy with the Introduction because the description of the tests is replicated. Delete it from the Introduction.

*Thank you for the comment. We deleted the redundant parts from the Introduction.*

The Discussion is well organized, but I have to underline the main concern of the paper: as you wrote, the 60% of the sample has a poor quality of the handstand and only 4 participants showed a very good quality.

*Although the sample originated from a pool of students with athletic backgrounds who passed basic gymnastics courses practicing handstands, their performance was lower. A better description of the sample selection rationale and a discussion about its limits are provided throughout the new manuscript (see Page 6, lines 2-9).*

In my opinion your paper has a very interesting starting point of view, but the tested sample has a very poor quality in performing handstand. Therefore, I suggest to deeply modify the target of your paper: you should direct your paper to novice athletes. If you don't do this the paper makes no sense, exactly because the quality of the motion is very poor. With this kind of modification, I will consider the paper for the publication.

*Thank you for the appreciation of our work and the suggestion on how to modify it. We followed the suggestion and focused the article‘s scope on prospective PE teachers and novice athletes.*

# Reviewer 2

Introduction and method sections were too long.

Need to highlight and summarize the main findings, with clear direct practical recommendation.

*Thank you for the suggestion. We rearranged and shortened the introduction section (see Pages 3-5) and edited the Methods section to streamline the text. We believe the current version better summarizes the study. We also provide a practical recommendation in the last paragraph of the Discussion (see Page 18, lines 1-4).*

Page 2, line 5-10: Add the characteristics of sample. Define each independent and dependent variable.

*Thank you for the suggestion. The new abstract now includes brief characteristics of the sample, and dependent and independent variables were defined (see Page 1, lines 6-11).*

Page 2, line 10-12: Show clearly the results with numerical findings support. Where is the conclusion?

*We added the numerical results and conclusion to the abstract (see Page 1, lines 12-18).*

Page 3, line 16: change “couches” to “coaches”

*Thank you for spotting this typo. We have corrected the error and proofread the text again (see Page 3, line 22).*

Page 4, line 8-9: convert references to numerical style.

*Thank you for noticing this. We converted these references according to PLOS ONE style guidelines.*

# Reviewer 3

The idea of the study is interesting, but the rationale got lost in the long Introduction. I recommend focusing the Introduction in few topics: 1) the importance of the handstand, such as presented in the first paragraph; 2) briefly what is known about shoulder role in the handstand execution; 3) why it is important to investigate the association of the tests with the handstand position, i.e., how could that help professionals of human movement in their practice or future research?

*Thank you for your very constructive feedback on our work. Following your suggested outline, we restructured and rewrote the Introduction to better focus the text. You can find all the changes highlighted (see Pages 3-5).*

"Data from this study are part of a larger project investigating the associations between the function and stability of the shoulder joint and the handstand execution quality." – I am concerned which other data have been investigated and why they have been presented separately.

*We agree that this statement may be misleading. It was meant to reflect our long-term study interest in various aspects of handstands pursuit in our team's multiple separate and individual studies. To amend the text, we have removed mentions of any other project.*

Table 1 – I suggest that a table with outcomes is presented in the results section and that sample characterization (age, weight and height) is presented in the text, using only mean and standard deviation.

*Thank you for the suggestion. We moved the table containing outcomes into the results section (see Page 12, line 22 and Page 13, line 1-3). We now report sample characteristics in the text (see Page 5, lines 21-22). We also added a supplementary table with mean results in the online supplemental material.*

Page 8, line 6: "The approximately 20 (±5) participants were divided into six groups." – please, clarify that. Do you mean the participants were divided into six groups of 20 participants each?

*We agree that the original information was vague. We have clarified this section, now it reads as follow: The participants were divided into six groups of 20. (see Page 6, line 15).*

p.8; l. 17-18 – why haven't you analyzed these data in the present study? They may be important predictors, I don't understand why splitting the analyses. Furthermore, if not using arm length information, why describing how it was obtained in the methods?

*The arm length is a needed variable for the UQYBT score calculation. We have rephrased the relevant section to clarify this (see Page 6, lines 23-24).*

The Sit-position test reliability and validity have been previously demonstrated? What about the use of Kinovea to assess it? Which parameters have been used for alignment assessment? Are they valid and reliable?

*Thank you for the comment. As the SPT is not a standardized test and the assessment was not done using Kinovea (or similar), we decided to remove the SPT from the article as it is not a relevant metric.*

I do not see a reason to investigate correlations between AQV and E-score or between CKCUES and YBT. Hence, I recommend removing the second and third paragraphs of the discussion section.

*Thank you for the comment. We removed the correlation between AQV and E-score from the text. We also removed the correlation between CKCUEST and UQYBT from the main text and moved it into the online supplemental material. Other authors report these two measures as positively correlated, but we did not observe such associations in our sample. The new version of the Discussion section only briefly mentions it (see Page 16, lines 3-8).*

The discussion section is confusing and does not focus on the main objective of the study. It sounds like the sample of the study was not adequate for investigating the hypothesis.

*Thank you for the comment regarding the focus of the Discussion. We rewrote multiple sections of the Discussion to provide better clarity and improve focus. The Discussion now includes a more developed section about our study sample and its limits (see Pages 15-18).*
