# Supplementary material for: Are the shoulder joint function, stability, and mobility tests predictive of handstand execution?
Source: PLoS One. 2024 May 13;19(5):e0302922. doi: 10.1371/journal.pone.0302922 (PMC11090318; doi:10.1371/journal.pone.0302922)
Supplement: Attachment — Submitted filename: Response to Reviewers.docx [file pone.0302922.s006.docx]

Dear Dr, Shaheen,

Thank you for sharing your comments and comments from the two reviewers regarding our manuscript submitted to the PlosONE journal. We believe the provided feedback helped us rework our manuscript into a stronger, better-focused piece. As you have suggested, we are resubmitting the improved version of our manuscript for review to the PlosONE again.

Below are individual comments and related responses (in italics) on how we have amended the manuscript.

# Reviewer 1

The introduction is based on citations of old publications, i.e. 1, 4, 9, 10, 11, 16, 23, 29, 30, 31, 33, 35. Please rewrite the introduction, and discuss these issues in the introductory part by referring to the following article: Puszczałowska-Lizis E, Omorczyk J. The level of body balance in standing position and handstand in seniors athletes practicing artistic gymnastics. Acta of Bioengineering and Biomechanics 2019; 21 (2): 37-44. DOI: 10.5277/ABB-01352- 2019-02.

Thank you for your observant comment and for sharing the reference we have missed in our literature review. We amended the introduction, and the Puszczałowska-Lizis et al. (2019) is included in the new manuscript (see page 3, line 24). Although we agree some of the cited studies are older, we are unaware of other publications related to the topic.

The study lacks a clear aim and research questions.

Thank you for this feedback. We have rewritten the final paragraph of the introduction to clearly state the study aims, research question, and predictions (Page 4, Lines 11-14).

Please provide the sample size calculation.

What was the method of random selection? Or was this a convenience sample?

A clarification of the selection criteria would be required.

*Thank you for the comment. The study is based on a convenience sampling. We recruited all available students on the faculty who met our inclusion criteria and were willing to participate voluntarily. An a priory sample size calculation was not conducted. The related section of the Methods is rephrased to better reflect the sample size rationale; see Page 5, Lines 2-14.*

Medians should be provided in the tables.

Thank you for the comment. We added medians into Table 1 as suggested; see page 12, line 3.

In the discussion, the authors present the results in the form of numerical data, which should not be the case. The Discussion is rather superficial and did not offer sufficient explanations for the mechanistic reasons behind the findings. You should add some limitations of the study.

Thank you for your feedback. We rewrote several segments of the Discussion to interpret the observed results better. We decided to provide numbers (e.g., effect and sample sizes) for easier objective comparisons of our results with other related studies. In our opinion, a purely qualitative assessment might lack some needed nuance.

The work lacks clearly formulated conclusions. Please specify the conclusions in points as answers to the research questions.

Thank you for your comment. We have added a separate paragraph in the introduction with clearly specified research aims. We also amended the Discussion section, which now includes a section concerning the study's main limitations and summarizes conclusions with clear answers to the research aims and questions.

The reference list contains too many old positions, i.e., 1,4, 9, 10, 16, 23, 29, 30, 31, 33, 35, 41. Please edit. References are quoted carelessly, with errors – please correct them.

Thank you for your feedback. We updated and corrected in-text citations and the reference list as per PlosONE citation style (see References pages 18-22).

# Reviewer 2

1) The introduction is too long, must be rewritten to focus on main aspects of the manuscript.

Thank you for your comment. We have reworked the introduction and removed superfluous paragraphs to focus the text better. We hope that the current introduction is more concise and more straightforward.

2) Page 3, line 19. The presented paragraph is redundant, it includes description that belong to the method section.

Thank you for your comment. We have moved and incorporated the entire paragraph into the Methods section, as suggested; see Page 7, Lines 19-24, and Page 8, lines 1-6.

3) Page 3 line 22, wrong method of reference, also this sentence while is related to handstand, press handstand is different gymnastic elements and should not be discussed in this manuscript.

We agree that this sentence is superfluous for the topic of our study. We have removed the sentence as suggested.

4) Page 4 line 1, wrong reference method, this occurs also in discussion section again.

Thank you for the observation. We have corrected all citations to match the PlosONE reference style requirements.

5) Page 7,9: please write “same investigators” or similar instead of Authors initials.

We have removed the authors' initials and substituted them with “members of the authors’ collective” in appropriate places; see page 6, line 7, and Page 8, line 7.

6) Page 8, The AQV is not clearly described, it is hard to follow, what will be best outcome and what will be the worst (failed to handstand), please indicate what was the maximum of point that participant could achieved due to the errors.

The text on page 7 (lines 12-18) now describes the AQV assessment in more detail. The AQV is a 5-point scale where "0" represents the worst (poor performance) and "4" represents very good performance with no errors.

7) Page 9 “arm length times three” it should be rewritten (for example multiplied) as it is misleading.

Thank you for your comment. We have replaced the term "times" with "multiplied" (see Page 8, line 24).

8) Page 9: I believe that “single arm push-up with legs a pelvic width apart” it is not correct description for this position, as the push-up implies flexing and extending the elbow joints in supporting arm. I recommend something like, single hand front support or similar.

We agree that the description of the position was inaccurate. We have rewritten the description as suggested ("single arm front support"; see Page 8, lines 15-17).

9) Why was the quality of handstands evaluated with two methods? What was the purpose? Each additional testing with similar variable increases potential bias.

Thank you for your comment.

We chose these two rating scales as they provide different insights into the execution quality. Although the AQV is the official FIG rating scale, it is limited in terms of resolution. While not an official rating scale, the E-score allows for a much finer evaluation.

Though these two scales were statistically significantly correlated (as can be expected) in our study, the strength of association was below a level (see page 13, lines 4-5) at which we would consider them interchangeable. Therefore, both provide separate insights into execution quality.

We have amended the related sections of the text to reflect better our rationale for using both scales; see page 7, lines 12-24, and page 8, lines 1-10.

10) Lower part of Table 1. Is vague and hard to follow, please change to make it clear.

Thank you for your feedback. We have redesigned Table 1 to make it more straightforward and accessible (see page 12, line 3).

11) There is no figure 2 introduced in the manuscript.

Thank you for noticing. We have corrected the image numbering throughout the manuscript.

12) While I understand the purpose of describing entire evaluation process, the details like division into 6 groups, the station approach etc. are non-important for the aim of the study. Please simplify this as it diminishes the clearance of the manuscript.

We agree, and we have thus removed this redundant information from the manuscript. See the rewritten text on page 5, lines 18-21.

13) Page 15 lines 1-2, There are missing symbols in the equations.

Thank you for noticing. We have added degrees of freedom to the calculation reporting (see page 14, lines 5-6).

14) While the Authors recognized the different shoulder position in their testing and handstand (90° vs 180°), more should be elaborated on the anatomy and function of shoulder joint as it comprises of glenohumeral joint and acromioclavicular joint.

Thank you for your comment. We have added a new section discussing the key differences in loading of the rotator cuff muscles in different ranges of shoulder flexion; see page 15, lines 19-25, and page 16, lines 1-2).
