# Supplementary material for: Are the shoulder joint function, stability, and mobility tests predictive of handstand execution?
Source: PLoS One. 2024 May 13;19(5):e0302922. doi: 10.1371/journal.pone.0302922 (PMC11090318; doi:10.1371/journal.pone.0302922)
Supplement: Attachment — Submitted filename: Response to Reviewers.docx [file pone.0302922.s007.docx]

Dear Dr Shaheen,

Thank you for sharing your comments and those of the two reviewers regarding our manuscript submitted to the PlosONE journal. We believe the feedback provided helped us rework our manuscript into a stronger, better-focused piece. As you have suggested, we are resubmitting the improved version of our manuscript for review to PlosONE again.

Below are individual comments and related responses (in italics) on how we have amended the manuscript.

# Reviewer 1

1) The study still lacks research questions. Please present research questions in bullet points.

Thank you for your comment. We added specific scientific question related to the aim of the study (see page 4, lines 11-17).

2) Please describe in more detail the data in Table 1.

We agree that the Table 1 was not sufficiently clear. We added additional detailed descriptions of the variables included in the table and better specified the note of the table (see page 11, lines 12-18)

3) Reference numbers: 22, 24 contain errors. Please proofread and cite these items according to Plos One requirements as follows:

Omorczyk J, Bujas P, Puszczałowska-Lizis E, Biskup L. Balance in handstand and postural stability in standing position in athletes practicing gymnastics. Acta Bioeng Biomech. 2018; 20 (2): 139-147. doi: 10.5277/ABB-01110-2018-02.

Puszczałowska-Lizis E, Omorczyk J. The level of body balance in standing position and handstand in seniors athletes practicing artistic gymnastics. Acta Bioeng Biomech. 2019; 21 (2): 37-44. doi: 10.5277/ABB-01352-2019-02.

Thank you for noticing; we corrected both references (see page 19, lines 6-8 and 12-14).

# Reviewer 2

1) Previous 9) “Why was the quality of handstands evaluated with two methods? What was the purpose? Each additional testing with similar variable increases potential bias. Thank you for your comment. We chose these two rating scales as they provide different insights into the execution quality. Although the AQV is the official FIG rating scale, it is limited in terms of resolution. While not an official rating scale, the E-score allows for a much finer evaluation. Though these two scales were statistically significantly correlated (as can be expected) in our study, the strength of association was below a level (see page 13, lines 4-5) at which we would consider them interchangeable. Therefore, both provide separate insights into execution quality. We have amended the related sections of the text to reflect better our rationale for using both scales; see page 7, lines 12-24, and page 8, lines 1-10.”

@While Authors improve the rational for the usage of two scores, still it exceeds the purpose of this manuscript. I see and recommend two options: 1) stay with only one score method as It was suggested previously, or 2) incorporate the issue of scoring methods in handstand to the manuscript and thus change the manuscript topic and introduction, aim, discussion etc. accordingly.

Thank you for your feedback. We adopted the first option and kept only the E-score as a handstand quality evaluation metric. We removed the AQV from the manuscript.

2) Page 8. Lines 12-14. This sentence should be earlier not in the description of the stations.

Thank you for your suggestion, we have moved the sentence (see page 6, lines 15-17).

3) Figure 3 and 4. There are no mention about them in the main text, they should also have the abbreviations explained in the figure caption.

Thank you for noticing. These were only remnants of previous manuscript revision, we forgot to delete the image captions. Only Figures 1 and 2 and their corresponding captions appear in the manuscript.

4) previous 10) “Lower part of Table 1. Is vague and hard to follow, please change to make it clear. Thank you for your feedback. We have redesigned Table 1 to make it more straightforward and accessible (see page 12, line 3).”

@Still the table is not so clear to the AQV description and again the abbreviations should be explained in tables.

We agree that the Table 1 was not sufficiently clear. We added additional detailed descriptions of the variables included in the table and better specified the note of the table (see page 11, lines 12-18)

5) previous 12) “While I understand the purpose of describing entire evaluation process, the details like division into 6 groups, the station approach etc. are non-important for the aim of the study. Please simplify this as it diminishes the clearance of the manuscript.

We agree, and we have thus removed this redundant information from the manuscript. See the rewritten text on page 5, lines 18-21.

@Still there are too much information on the procedure, that is not related directly to the outcome. Now, the Authors write about six groups of 20 so there was 120 participants? Materials describe 111. The indicating of stations it is unnecessary and blurs the image of research.

We removed this redundant and misleading information and streamlined the text (see page 5, lines 18-22).

6) previous 13) Page 15 lines 1-2, There are missing symbols in the equations.

Thank you for noticing. We have added degrees of freedom to the calculation reporting (see page 14, lines 5-6).

@I believe that there should be “􀀀2” but in PDF version I still see only blank squares.

Thank you for noticing. To avoid errors of character transcoding, we have replaced the χ^2^ with ‘chi-squared’ (see page 13, lines 3-5).

7) previous 14) While the Authors recognized the different shoulder position in their testing and handstand (90° vs 180°), more should be elaborated on the anatomy and function of shoulder joint as it comprises of glenohumeral joint and acromioclavicular joint.

Thank you for your comment. We have added a new section discussing the key differences in loading of the rotator cuff muscles in different ranges of shoulder flexion; see page 15, lines 19-25, and page 16, lines 1-2).

@While the description improved, in my opinion this part focuses only on the glenohumeral joint and no elaboration on acromioclavicular/sternoclavicular joint is made.

Thank you for the comment. We tried to develop the related discussion section covering all joints of the brachial plexus. However, we are unaware of any additional available literature regarding specific articulations of the brachial plexus (especially acromioclavicular/sternoclavicular joint) concerning handstands or single-joint tests.

See the amended discussion section on page 15.

8) Table 2 and 3 should have their captions above the table not underneath.

We moved the captions above the tables (see page 13, line 5).
